# Genome Sequence of *Proteus mirabilis* Strain PR03, Isolated from a Local Hospital in Malaysia

**DOI:** 10.1128/genomeA.00327-13

**Published:** 2013-06-06

**Authors:** M. I. H. Khalid, L. K. Teh, L. S. Lee, Z. A. Zakaria, M. Z. Salleh

**Affiliations:** Pharmacogenomics Centre (PROMISE), Faculty of Pharmacy, Universiti Teknologi MARA (UiTM) Malaysia, Selangor Darul Ehsan, Malaysiaa; Department of Biomedical Science, Faculty of Medicine and Health Sciences, University Putra Malaysia (UPM), Selangor, Malaysiab

## Abstract

*Proteus mirabilis* is one of the pathogenic agents that commonly causes urinary tract infections among elderly individuals and long-term catheterized patients. Here, we report a draft genome sequence of *Proteus mirabilis* strain PR03 (3,932,623 bp, with a G+C content of 38.6%) isolated from a local hospital in Malaysia.

## GENOME ANNOUNCEMENT

*Proteus mirabilis* is a Gram-negative bacillus, commonly isolated from patients with urinary tract infections or long-term catheterization (1). Urinary stones formed by *Proteus mirabilis* block urinary catheters of the patients (2). Members of this species have dimorphic morphologies and are motile. In liquid medium, the species appeared as a short bacillus (1 to 2 µm), while in medium with low viscosity the bacilli became elongated (10 to 80 µm) (3).

Here, we report a draft genome sequence of *Proteus mirabilis* strain PR03. The isolate was isolated from a patient in a local Malaysian hospital. Sequencing was performed using Genome IIx (Illumina, San Diego, CA) at the Pharmacogenomics Centre (PROMISE), Faculty of Pharmacy, Universiti Teknologi MARA (UiTM), Malaysia. A total of 8.89 gb of short reads was produced, with a deep coverage of 605-fold. The short-read quality was determined using FastQC and CLCBio WorkBench version 6.0. A total of 73 contigs were produced using CLCBio *de novo* assembly. OSLay software (4) was used to assemble the contigs into 4 scaffolds for the draft genome of this *P. mirabilis* strain PR03. The draft genome was viewed using Artemis software (5). The genome was annotated using Rapid Annotation Subsystem Technology (RAST) (6) and validated by the Bacterial Annotation System (BASys) (7).

The circular draft genome was comprised of 3,932,623 bp with a G+C content of 38.6%. A total of 3,465 coding sequences (CDS) and 53 tRNA regions were annotated. Of the CDS, 2.35% were associated with virulence, diseases, and defenses; 6.33% were associated with the cell wall and capsule; 4.72% were related to the stress response; and 2.02% were related to clustered chemotaxis and motility genes. The clustered motility genes observed in a previously reported strain, *P. mirabilis* HI4320, were also observed in the current *P. mirabilis* strain PR03. This strain is predicted to be resistant to tellurite, arsenic, tellurium, tetracycline, quaternary ammonium compounds, methyl viologen, albacidin, acriflavine, bicyclomycin, and fluoroquinolones.

### Nucleotide sequence accession numbers.

The genome sequence of *P. mirabilis* strain PR03 has been deposited at DDBJ/EMBL/GenBank under accession no. AORN00000000. The version of the genome described in this paper is the first version (GenBank accession no. AORN01000000).
